# Genetic investigation into an increased susceptibility to biliary atresia in an extended New Zealand Māori family

**DOI:** 10.1186/s12920-018-0440-0

**Published:** 2018-12-18

**Authors:** Sophia R. Cameron-Christie, Justin Wilde, Andrew Gray, Rick Tankard, Melanie Bahlo, David Markie, Helen M. Evans, Stephen P. Robertson

**Affiliations:** 1 Department of Women’s and Children’s Health, Dunedin School of Medicine, University of Otago, Dunedin, 9054 New Zealand; 2 Department of Paediatrics, Tauranga Hospital, Tauranga, New Zealand; 3 Department of Preventive and Social Medicine, University of Otago, Dunedin, 9054 New Zealand; 4 Population Health and Immunity Division, The Walter and Eliza Hall Institute of Medical Research, 1G Royal Parade, Parkville, VIC 3052 Australia; 5 Department of Pathology, University of Otago, Dunedin, 9054 New Zealand; 6 Paediatric Gastroenterology and Hepatology, Starship Children’s Health, 2 Park Road, Grafton, Auckland 1023 New Zealand; 7 Department of Medical Biology, The University of Melbourne, Parkville, VIC 3010 Australia

**Keywords:** Biliary atresia, Exome sequencing, Population genetics, Paediatric disease

## Abstract

**Background:**

Biliary atresia (BA), a fibrosing disorder of the developing biliary tract leading to liver failure in infancy, has an elevated incidence in indigenous New Zealand (NZ) Māori. We investigated a high rate of BA in a group of children (*n* = 12) belonging to a single Māori iwi (or ‘tribe’, related through a remote ancestor).

**Methods:**

Population and geographical data was used to estimate the rate of BA in Māori sub-groups, and a pedigree linking most of the affected children was constructed from oral and documented history. Array genotyping was used to examine hypotheses about the inheritance of a possible genetic risk factor, and the history of the affected population, and Exome Sequencing to search for candidate genes.

**Results:**

Most of these affected children (*n* = 7) link to a self-reported pedigree and carry a 50-fold increase in BA risk over unrelated Māori (χ^2^ = 296*P <* 0.001, 95% CI 23–111). Genetic analysis using FEstim and SNP array genotypes revealed no evidence for elevated consanguinity between parents of affected children (FEstim: *F* (2,21) = 0.469, *P >* 0.63). Genome-wide quantitation of intervals of contiguous, homozygous-by-state markers reached a similar conclusion (*F* (2,399) = 1.99, *P* = 0.138). Principal component analysis and investigation with STRUCTURE found no evidence of increased allele frequency of either a recessive variant, or additive, low-risk variants due to reproductive isolation. To identify candidate causal factors, Exome Sequencing datasets were scrutinised for shared rare coding variants across 8 affected individuals. No rare, non-synonymous, phylogenetically conserved variants were common to 6 or more affected children.

**Conclusion:**

The substantially elevated risk for development of BA in this subgroup could be mediated by genetic factors, but the iwi exhibits no properties indicative of recent or remote reproductive isolation. Resolution of any risk loci may rely on extensive genomic sequencing studies in this iwi or investigation of other mechnaisms such as copy number variation.

**Electronic supplementary material:**

The online version of this article (10.1186/s12920-018-0440-0) contains supplementary material, which is available to authorized users.

## Background

Biliary atresia (BA) is a phenotype of unknown, likely complex, aetiology characterized by congenital, fibrotic obliteration of bile ducts that evolves over the late fetal and early infant periods of development. It is the most common reason for paediatric liver transplant referral [1] and leads to liver failure and death without early surgical intervention [2]. BA usually presents as an isolated clinical phenomenon but can be a component of a syndromic entity with major malformations [3] and biliary cysts [4], leading to the classification of the disease into multiple categories [5, 6]. The heterogeneity of its presentation in a variety of syndromes, and the inconsistency of correlative factors between different studies, suggests it is not likely to be a single disease with a unifying aetiology, but more a phenotype with multiple contributing factors converging on a similar pathology [5]. ‘Isolated BA’, when BA occurs in isolation from other non-hepatic or biliary symptoms, is the most common form of the disease [7]. The influence of environmental factors is implied by possible correlations between seasonality and disease onset in some cohorts [8–14] (although evidence is lacking in others [7, 15–22]), putative associations with viral infections [23–26], the isolation of a plant toxin that causes the disease in livestock of multiple species [27, 28], and evidence of greater risk in non-urban children [8] and those resident in regions with lower population density [8]. Genetic factors have also been implicated in isolated BA, including a chromosome 10q24.2 region identified by Genome Wide Association Studies (GWAS) in both SE Asian and European populations with an odds ratio of 1.45–1.77 [29–31]. However, no highly penetrant genetic factors involved in the isolated form of the disease have been identified to date.

There are well-replicated differences in the prevalence of BA, both between ethnically different populations [12, 21, 32–35], and between ethnic groups living within the same region [36, 37], and the established predisposition to this disease in New Zealand Māori which was re-evaluated recently (author HME, *In press,* [38]). Elevated rates are found in certain populations across different countries, climates and socio-economic contexts, with particularly high rates in East-Asian [11, 21, 22, 32, 39–43] and Polynesian groups [14, 18, 35, 44], including New Zealand Māori [38]. A difference in response to treatment has also been observed in African-Americans [45], who also have a higher rate of BA than European Americans [9, 46]. Together these data suggest that ethnicity-specific, possibly genetic, risk factors influence the incidence and course of this disease.

Māori are the indigenous population of New Zealand, descended from Polynesian explorers c.750 years ago [47]. During the migration of large numbers of Europeans in the early nineteenth century, the Māori population declined precipitously due to introduced diseases and conflict [48, 49]. Subsequently the population has grown to over 600,000 people, living in NZ and overseas. There has been substantial admixture with post-colonial settlers of mainly European descent [50], although approximately half of those who identify as Māori do not also identify with another ethnicity [51]. Studies three decades ago reported a 10-fold increase in the rate of BA in Māori relative to NZ Europeans [52]. Over the last two decades, mandatory referral of all cases of BA to a national centre for treatment (Starship Children's  Health, Auckland) has facilitated the recalculation of the risk for this disorder amongst Māori to approximately 3-fold over that observed in NZ Europeans [38]. This recent study also found that BA in children with Māori ancestry is qualitatively different from those of European-only descent, in that Māori children with BA are more likely to be effectively palliated by the Kasai procedure, and hence develop a requirement for liver transplant much later.

An iwi (often translated as “tribe” but also the less structured “people” [53]) is the largest unit of Māori society operating as both a social and legal distinction. Identification with an iwi is a cultural connection that also signifies common biological ancestry [49] back to the original Polynesian founders of New Zealand [54]. This ancestry is recorded by Māori through detailed whakapapa (‘genealogy’) as a fundamental aspect of their culture. Māori membership of an iwi, along with smaller societal units such as hāpu (‘clan’) and whanau (‘extended family’) are therefore of genealogical relevance. However, Māori social organisation has been dynamic and adaptive prior to, and after European colonisation [53, 55]. Historical evidence shows significant geographical movement and intermarriage of the communities who now identify as iwi [56, 57]. This paper investigates BA in two New Zealand iwi, with shared history and intermarriage (referred to as a single iwi here), named Ngāiterangi and Ngāti Ranginui. A much greater number of BA cases than expected, based on NZ prevalence rates, has been reported in this iwi over the last twenty-five years.

We hypothesise that the elevated rate of BA in the iwi is due to a genetic susceptibility. We show that the iwi as a whole does not exhibit characteristics of reproductive isolation, making it unlikely that a major shift in the allele frequency of many low-risk alleles could have occurred by drift to confer a susceptibility to the development of BA. These observations are congruent with a model that proposes that a dominantly-acting, low-penetrance susceptibility factor could explain this cluster of BA cases.

## Methods

### Ethics

All subjects were ascertained by physician-initiated referral and consented to participate under a protocol approved by the Southern Regional Ethics Committee (13/STH/56).

### Patient ascertainment and ethnicity

Patient information was ascertained from the national registry of BA patients and curated by one of the authors (HME). Patients were classified as Māori following interview to ascertain a prioritized ethnicity, a hierarchical system commonly used in NZ to attribute a single ethnicity to those with multiple contributing ethnicities. In this system, Māori ethnicity is classified as the highest hierarchy. Mothers of affected iwi individuals retrospectively self-reported their health status and any medical issues of note during the course of their pregnancies.

### DNA genotyping and sequencing

ES was performed and SNP array data was genotyped for the following individuals within the iwi (see iwi description below): all living, affected individuals (J-5 to J-7 and I-1 to I-5) and their parents (with the exception of the father of J-5), the I-1 sibling with a choledochal cyst, the mother of J-1, and the parents of J-4. DNA extraction from blood was performed using Wizard® Genomic DNA Purification Kit from Promega. FFPE DNA extracted by Zymo FFPE DNA miniprep and Zymo purification kit (ZR-96 DNA Clean & Concentrator™-5) was used for FFPE samples. Genotyping was performed using the Omni1-Quad and Omni5 chip platforms (Illumina) and Exome Sequencing (ES) (Roche SeqCap EZ2, Agilent SureSelect Human AV4, and AV4 + UTR Exon capture chips) was performed by Otogenetics (Atlanta, GA, USA) on the Illumina HiSeq 2000 platform.

FASTQ files were aligned, processed and called on a pipeline consistent with the Genome Analysis Toolkit (GATK) Best Practices [58], and used BWA-MEM [59] for alignment, Picard for sorting and marking duplicates, and GATK IndelRealigner for indel realigning followed by base quality score recalibration and GATK’s Haplotypecaller for individual variant calling followed by joint genotyping with GenotypeGVCFs to produce a multi-sample Variant Call Format (VCF) file. Variant annotation performed with SNPeff [60]. Over the patient group, variants were fitted to either a monoallelic (dominant) model or a biallelic (recessive) model.

### Population data

Rates of BA across the entire iwi were determined using population estimates from census data of New Zealanders citing membership of the iwi who were born from 1988 onwards, a period for which census and national BA data were available and which incorporates the period during which the iwi children with BA were born. Population size estimates for affected Pedigree J individuals (see iwi description below) were estimated as ~ 850 people born in the last 30 years, based on historical records of founder pair J’s children and the growth of the Māori population since the birth of those children (Additional file 1: Table S1).

### Software

STRUCTURE and EIGENSOFT’s Principal component analysis (PCA) were used to investigate population structure in the samples. STRUCTURE is an algorithm that uses Bayesian clustering to both estimate allele frequencies for a given number of populations, and assign samples to those populations, without a priori information about the samples’ expected groupings. STRUCTURE was run iteratively over 10 populations, each with 5 iterations and random seeds. StructureHarvester [61] was used to determine the most likely number of discrete populations (K) across the whole cohort using data generated by STRUCTURE [62]. CLUMPAK [63] was used to summate and visualise the data for the selected K-value (population number). EIGENSOFT uses PCA to define discrete patterns across many dimensions of data (in this case, > 7000 genetic markers). Germline [64] was used to call runs of markers for homozygosity analysis. Allele frequencies used in FEstim [65] were estimated using Plink [66] from 28 unrelated Māori.

### Statistical analysis

R Software [67] was used for statistical analysis of FEstim and homozygous-by-state (HBS) data, while Stata 14.2 [68] was used for other statistical analyses including calculating relative risks. Incidence rate ratios were obtained from Poisson regression using calendar month or season (quartiles of January–March, April–June, July–September, and October–December) while adjusting for year as a continuous predictor. Probability that the risk of BA is altered by birth month was expressed using the Wald test [69, 70]. For one-sided statistical analyses (Chi-squared, Wald) *P <* 0.05 indicates threshold of statistical significance, for two-sided (two-sided T-tests), *P <* 0.025 indicates threshold of statistical significance (signficance *P <* 0.05 when accounting for both tails).

### Description of iwi cluster and pedigree structure

**Fig. 1:**
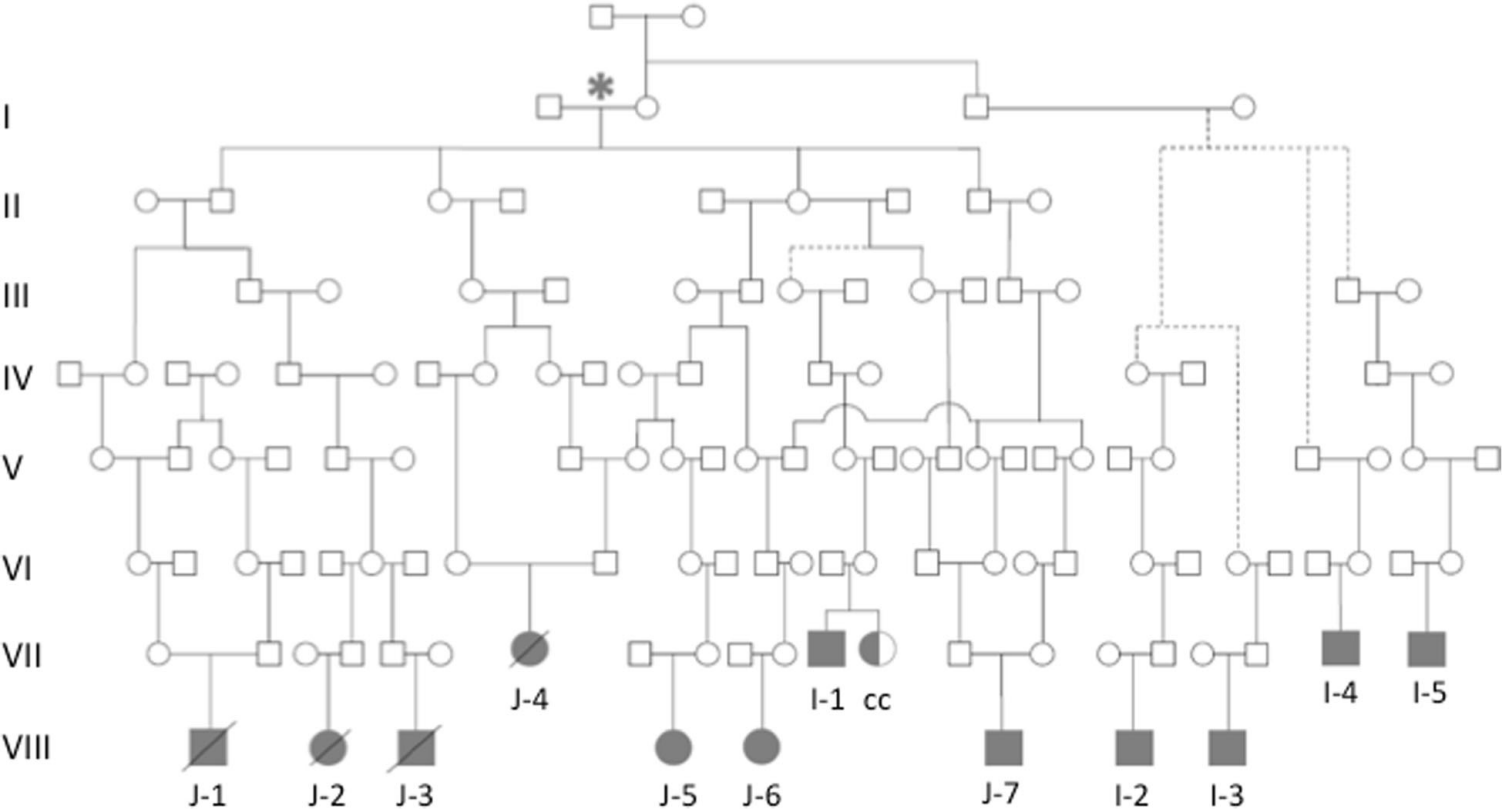
Pedigree of the twelve BA families from the iwi (twelve shaded individuals with BA, one half-shaded with a choledochal cyst). The birth dates for the children of the Pedigree J founders are in the mid to early nineteenth century. Affected individuals born after the year 2000 are shown in the bottom row while those before the year 2000 are in the row immediately above. Many other siblings and family members are not shown for brevity. Dotted lines represent instances where explicit, contiguous connections to the rest of the iwi via named individuals were absent, but the members reported kinship via older ancestors. Affected individuals in the J-group are labelled J-1 to J-7, while those from the wider iwi are I-1 to I-5. The sibling with a choledochal cyst is labelled as ‘cc’. Generation labels are given only for the J-Group, beginning with Couple J (indicated with an asterix)

**Fig. 2:**
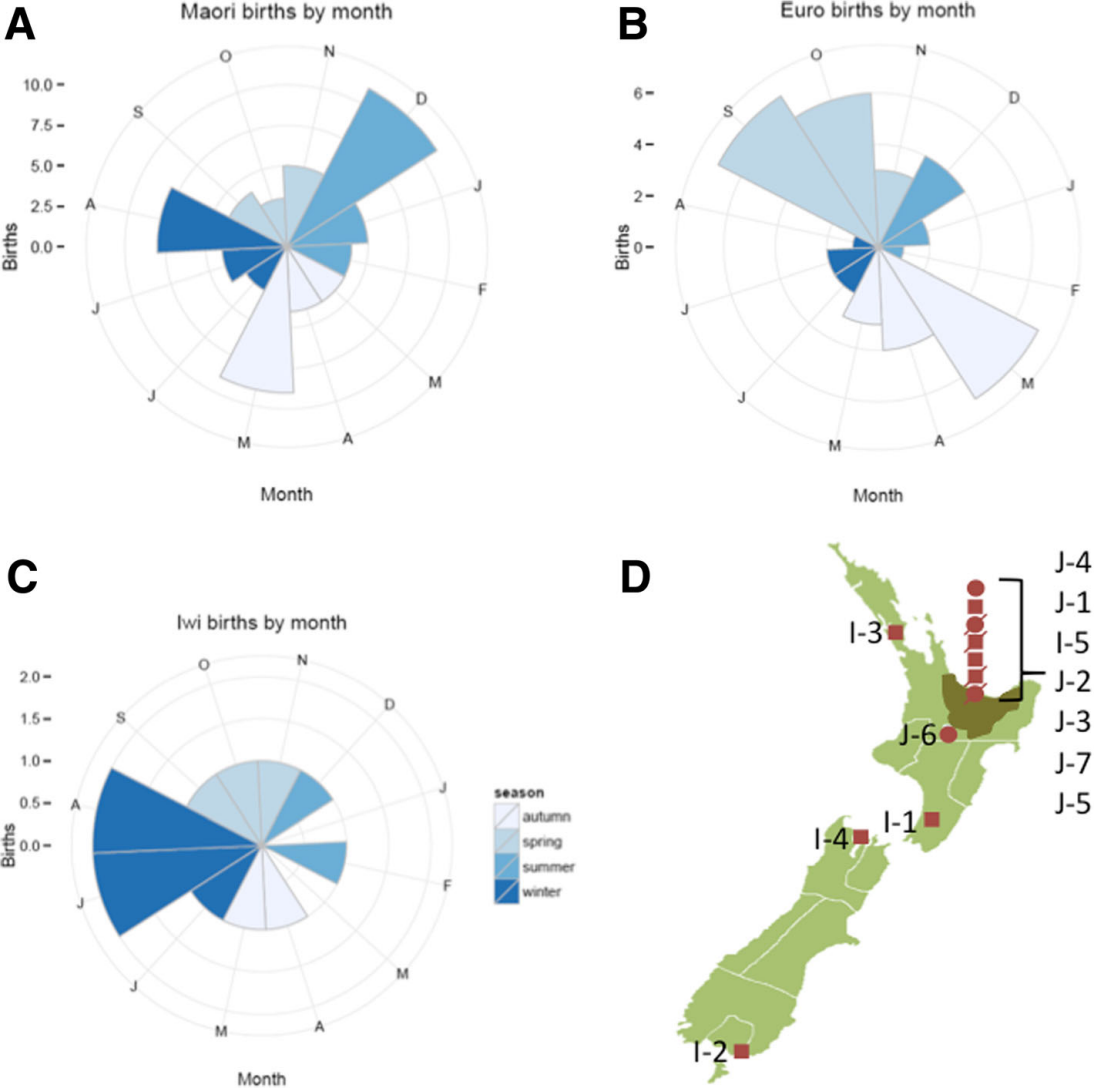
Rose plots indicating the rate of BA births by month for **a** Māori, **b** European and **c** Individuals from the iwi from New Zealand, with colours indicating the Southern Hemisphere seasons (see key on diagram). **d** Map of New Zealand with the Bay of Plenty region shown in dark green, and birth places of children shown in dark red (circles for female, squares for male, and strikethroughs for deceased individuals). NZ map adapted from LJ Holden, distributed under a CC-BY-SA 3.0 license

Over the period 1991–2009, incidental note was made of a high rate (*n* = 7) of BA in Māori children resident in the Bay of Plenty region of New Zealand, on the east coast of the North Island. Some family members were related to other historical BA cases from the area, and also to another affected child born elsewhere in New Zealand. These family members identified as part of two Bay of Plenty iwi, which will be referred to as ‘the iwi’ singular for brevity. At the 2013 census, 3.1% of all Māori identify as part of this iwi, as well as 11.6% of Māori who were resident in the Bay of Plenty [51], making them the 3rd and 5th most common iwi reported in this region. However, all the children born with BA in this Bay of Plenty cluster identified in this iwi except for one (I-5, Fig. 1 & Fig. 2), and this child reported close geographical and familial connections to other affected children. Five other affected individuals living elsewhere in NZ also had whakapapa connections to the iwi and were also born during the same time period.

Extensive whakapapa (recounting of lineage and familial relationships) by family members was used to construct a pedigree of the seven children with known relatedness (Fig. 1, J-1 to J-7), extending back seven generations to a Māori-European founder pair in the early nineteenth Century (Founder Pair J). This extended family (denoted ‘Pedigree J’ in this work) in the context of the wider pedigree is shown in Fig. 1. The remaining five affected individuals (I-1 to I-5) could not be explicitly linked to this pedigree by unbroken lines of ancestry but nevertheless can be considered to be related to the iwi with high confidence given the centrality of whakapapa and lineage to Māori culture.

This entire group of 12 affected individuals are therefore considered to be ancestrally connected. Eleven of these individuals had isolated BA, while one (J-2) was excluded from analysis because they presented with syndromic BA with co-existing complex congenital heart disease in the context of a history of poorly controlled maternal diabetes during pregnancy. They were excluded from genetic analysis, but were included in calculations of population incidence figures in keeping with previously published literature, as syndromic BA is not distinguished from non-syndromic presentations in most population-wide studies of the disorder. A thirteenth individual, who is the sibling of an individual with BA, underwent surgery for a congenital choledochal cyst (CC) but had no atresia of the bile ducts. This child was not included in calculations of BA incidence but had ES and array data collected. Apart from this instance there were no instances of recurrence of BA among 20 siblings of affected individuals (by 2009). Furthermore, there were no cases of BA reported from within the iwi or pedigree prior to 1991, even after extensive liaison and questioning with the extended family iwi kuia and kaumatua (elders) and their constituent whānau (extended families, friends and supporters).

## Results

The clinical observation of an apparently elevated rate of BA prompted us to formally calculate the incidence of BA in this iwi. The rate of BA was estimated relative to that observed in all Māori who did not identify as a member of the iwi. For this estimation, the affected individuals in the iwi were divided into two groups; those that could name shared ancestors through unbroken lineages (J-1 to J-7 or “Group J”), and a broader group that included the pedigree and also those who had connections to the iwi but could not cite them explicitly (I-1 to I-5). Census data from 2013 of living individuals born since 1988 was used as a denominator for estimating rates because these data covered the period of all affected iwi births and included iwi identification. No new BA cases had been reported in the iwi since the initial 2009 observation of the cluster. The population size of the affected generation descended from Couple J (Additional file 1: Table S1) was estimated by beginning with documented numbers of children and extrapolating their offspring at each subsequent generation from the population growth rates of Māori at each generation [48]. This assumes that an average of one in three ancestral couples were from within the same lineage based on the rate of intermarriage calculated empirically in the reported pedigree.

Excluding the affected individuals who identify as part of the iwi, 49 children with Māori ancestry were born with BA in the same period as the iwi BA cases, from a population ~ 30 times larger than the iwi population under study. Over the same period, 43 children of European ethnicity were diagnosed with BA. This translates to a rate of BA in the wider iwi of 1 in 903 live births, compared to a rate of 1 in 6110 in the general Māori population and 1 in 22,228 in the NZ European population. The incidence of the disorder in the central pedigree (Group J) as defined in Fig. 1 is even higher than the wider iwi, estimated at 1 in 121.

Relative rates are shown in Table 1, which shows the ratio of BA incidence in the iwi and sub-groups compared to relevant control populations; multiple ratios for the J-Group are shown, comparing them to the iwi and to the Māori population whether or not the iwi is included in this control group. Children who identify as a member of the iwi have a 7-fold greater risk of BA than Māori children in New Zealand who are not part of this iwi (RR 7, χ^*2*^_1_ = 44.2, 95% Confidence Interval (CI) 4–13, *P <* 0.001). However, Pedigree J individuals have a 19-fold greater risk than iwi children who are not part of the pedigree (RR 19, χ^*2*^_1_ = 42.69, 95% CI 6–64, *P <* 0.001) and a 50-fold greater risk of BA compared to non-iwi Māori (RR 50, χ^*2*^_1_ = 296, 95% CI 23–111, *P <* 0.001). In contrast, iwi individuals outside the central pedigree have a 2.7 times increase in BA rates compared to non-iwi Māori (χ^*2*^_1_ = 3.93, 95% CI 1–7, *P =* 0.048). As expected from previous reports on the incidence of BA in New Zealand, and in Asian and Pacific populations, Māori have a significantly higher risk of BA compared to Europeans (RR 4, χ^*2*^_1_ = 63.72, 95% Confidence Interval (CI) 3–6, *P <* 0.001).

**Table 1:** Relative risks (RR) of BA in query populations compared to control populations

Query population	Control population	Relative Rate
J-group Only (*n* = 7/843)	Iwi (excluding J-group) (*n* = 4/9079)	RR: 19 *P* < 0.001, χ2 = 42.69 95% CI 5–64
J-group Only (*n* = 7/843)	All Māori BA (including non-J-group iwi) (*n* = 53/308,415)	RR: 48 *P* < 0.001, χ2 = 284.19 95% CI 22–105
J-group Only (*n* = 7/843)	All Māori BA (excluding iwi) (*n* = 49/299,336)	RR: 50 *P* < 0.001, χ2 = 296.12 95% CI 23–111
Iwi (Including J-group) (*n* = 11/9922)	All Māori BA (excluding iwi) (*n* = 49/299,336)	RR: 7 *P* < 0.001, χ2 = 44.15 95% CI 4–13
Iwi (excluding J-group) (*n* = 4/9079)	All Māori BA (excluding iwi) (*n* = 49/299,336)	RR: 3 *P* = 0.048, χ2 = 3.93 95% CI 1–7

Mothers were retrospectively asked about illnesses or exposure to drugs and toxins during pregnancy, but no obvious infections, exposure to toxins or drugs (prescribed medication or non-prescribed substances) were reported during the pregnancies of the iwi BA cases. No mothers had clinically diagnosed autoimmune disorders. The majority of pregnancies took place in the Bay of Plenty, where 40–50% of the iwi currently live [51] but five of the twelve individuals (J6, I-1 to I-4) were born in other regions across New Zealand (Fig. 2).

In the absence of identifiable environmental factors being reported by the mothers of affected iwi individuals, an environmental link to the BA cluster was further sought focusing on two factors previously associated with the disorder in other populations, seasonality (which varies by study [8–14] [7, 15–22]) and a higher risk of BA in births outside high-density urban centres [16]. To investigate seasonality, the rate of births by month and season was compared within the ancestral groups: all Māori, iwi-identifying Māori and NZ European. As shown in Fig. 2, there was no evidence of differences by birth month for affected iwi individuals (Wald *P =* 1.00); nor was there evidence of monthly differences for Māori (Wald *P* = 0.92), Europeans (Wald *P* = 0.23) or all births combined (Wald *P* = 0.37). There was also no evidence of differences found for seasonal quartile, (see Methods), with Wald *P* = 0.98 for iwi, *P* = 0.99 for Māori, *P* = 0.61 for Europeans, and *P* = 0.29 for all births. Children with BA from the iwi were classified into those born inside or outside main urban areas, as defined by the New Zealand census [51]. Sample sizes are too small to consider a formal test: however, two individuals with BA (17%) in the iwi kindred were born outside of main urban areas compared to rates of 26% of Māori women of childbearing age recorded to be living outside main urban areas.

Since these affected individuals were born in widely dispersed geographical locations across New Zealand and a common environmental explanatory factor was not readily evident to explain the clustering of BA in this iwi, this led us to conduct a genetic study in this kindred.

Since consanguinity was explicitly reported between parents of three of the twelve affected individuals (J-1, J-4 and J-7), tests for homozygosity in the other affected iwi children were performed. Where DNA was available from living individuals (J-5 to J-7 and I-1 to I-5), SNP chip data from affected individuals were examined for signals of homozygosity that could indicate parental relatedness, to test whether those with no known parental consanguinity nevertheless showed elevated homozygosity. In affected iwi individuals, elevated homozygosity was investigated by comparison to their parents (representing a group of individuals from the same population, without BA) and a group of parents of children with BA but no connection to the iwi using both parametric (FEstim) and nonparametric methods (homozygousity-by-state). FEstim [65] estimates homozygosity across genome-wide markers using a hidden Markov model with the assumption of linkage equilibrium. FEstim homozygosity estimates were compared to the levels of expected homozygosity, given the allele frequencies from the broad population under study. Continuous runs of markers that are homozygous-by-state (HBS) were also identified within samples because larger segments could indicate more recent consanguinity. Although there is a decrease in both Identity-by-descent (IBD) segment size as measured in centiMorgans (cM) and number of segments with increasing numbers of meioses, relationships between parents as remote as 5th cousins are detectable using this approach [71].

One affected child (I-1) whose sibling had a choledochal cyst was expected to show little or no measurable homozygosity as one parent was of European ancestry and was born outside of New Zealand. It had been assumed *a priori* that their affected status would be unlikely to be explained by a homozygous recessive allele. As a result they were not included in the statistical comparisons below so as not to bias the affected group towards lower estimates of homozygosity.

**Fig. 3:**
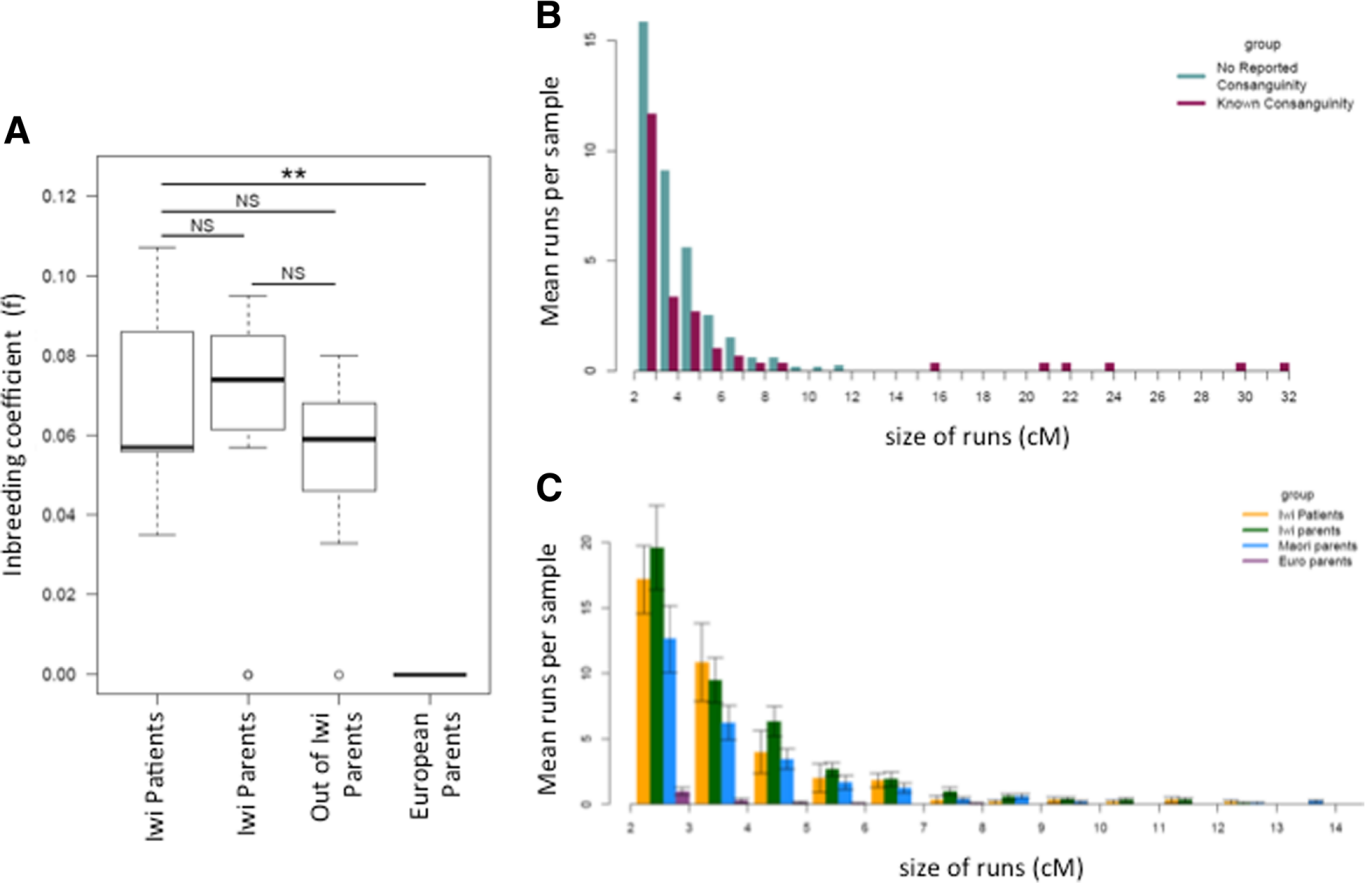
**a** Inbreeding Co-Efficients for Iwi BA Cases Compared to Parents, estimated using FEstim, and groups compared with ANOVA. **b** Rates of Homozygosity-by-State (HBS) runs of continuous markers in known Consanguineous Iwi Samples (*n* = 2) Compared to iwi parents Without Reported Consanguinity (*n* = 12). **c** Homozygosity Estimated by Size of HBS Runs. Groups: affected Iwi individuals (*n* = 6), parents of affected iwi individuals (*n* = 12), Māori parents of BA children with no reported connection to the Iwi (*n* = 14) and unrelated Europeans (*n* = 14). All had no known parental consanguinity

No significant difference was found in FEstim scores across the three Māori groups (affected children, iwi parents and Māori parents of affected children outside the iwi) (ANOVA, *F* (2,21) = 0.469, *P =* 0.63). Runs of HBS were compared between offspring of parents from within the iwi with known consanguinity, compared to those from iwi parents with no reported ancestral connection. Only those samples with reported parental consanguinity (J-7 and his father) were found to have runs of homozygosity over 11 cM (Fig. 3). Samples from children with BA without known parental consanguinity were then compared to the two unaffected parental groups (Fig. 3). Mean size of HBS runs did not differ between these three sample groups (ANOVA, *F* (2,399) = 1.99, *P =* 0.138). Of the five affected subjects without reported consanguinity (J-5 to J-6 and I-2 to I-5), only a single run above 11 cM was detected in one affected individual with no parental consanguinity, but whose parents both belonged to the iwi, possibly indicating distant consanguinity. Using both of these measures it can be concluded that individuals with BA in the iwi do not have elevated homozygosity compared to their parents or to Māori from outside the iwi.

Since an elevated rate of BA compared to other Māori has been identified only in this iwi (no clusters have been reported in other NZ areas or communities), a recessive variant whose allele frequency has become elevated by genetic drift would be more likely to be specific to the iwi if the population was genetically isolated. To test whether there is population structure (the presence of genetic diversity correlating to sub-populations) between members of the iwi and other Māori, all Māori parents for whom genotyping data were available (*n* = 28) were divided into those who were part of the iwi and/or pedigree J and those who were not. EIGENSOFT’s smartpca [72] and STRUCTURE [62] were used to investigate population differences between the two groups (see methods for a description of STRUCTURE’s application). Data from seven individuals of European ancestry were included to facilitate the detection of European admixture that was expected in most of the samples from Māori analysed in this study. The expectation was that admixed samples would cluster closer to Europeans in PCA and that such admixture would be accurately identifiable when using STRUCTURE.

**Fig. 4:**
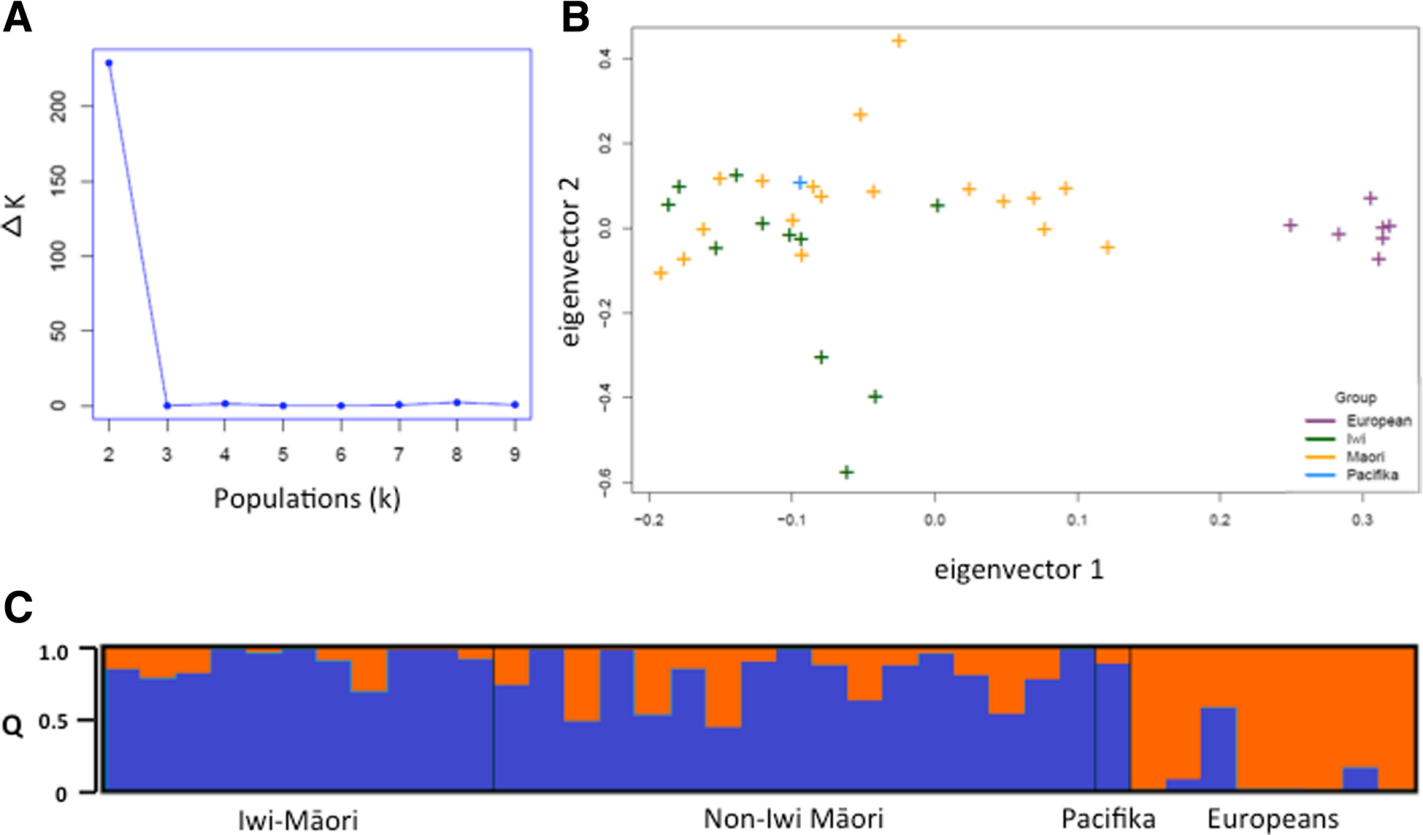
Results from smartPCA, STRUCTURE and StructureHarvester. **a** Delta K for Iterative Populations. The Evanno method estimated the most likely number of populations (K) as two, from STRUCTURE results. **b** PCA from founder samples using Eigenstrat. **c** Results of STRUCTURE with two inferred populations: blue correlates with Māori ethnicity, orange with European ethnicity

The first eigenvector identified in the dataset from the PCA analysis corresponded to European ancestry (Fig. 4). There was no significant difference between iwi samples and non-iwi Māori samples along the second eigenvector nor the subsequent vectors, such as would be expected if these groups exhibited substantial population structure. Two populations (correlating to Māori and European samples) were also shown to be the best hypothesis to explain the STRUCTURE data using the Evanno method through StructureHarvester [61]. Population structure was not detected in the Māori samples outside of the expected European admixture that was common across this entire group. The degree of European admixture between the in-iwi and out-of-iwi parents was not significantly different under a two-side t-test, in which 0.025 is classed as significant (Welch Two Sample t-test, *t* = 2.30, df = 26.49, *P =* 0.03) between these two populations. Three European samples were predicted to have Māori admixture; the highest (predicted admixture proportion of 0.58) was in a sample that was determined to have been mislabelled, and whose original records gave her ethnicity as Māori. One of the others, with 0.07 proportion of predicted admixture, was known to have two Māori great-great-great grandparents. The third unexpectedly admixed sample (predicted admixture = 0.16) remains unexplained, but as a New Zealand European, may have unreported Māori ancestry.

The unremarkable rates of homozygosity in affected children and the lack of evidence of an iwi-specific genetic bottleneck indicative of reproductive isolation, suggests that a founder effect leading to a single recessive factor is unlikely to explain a genetic component of BA susceptibility in the iwi. As an alternative, a model proposing a dominantly acting variant of low penetrance that could account for the 50-fold increase in BA within the pedigree was tested. As opposed to common alleles of minor effect that are often non-coding, variants associated with substantial influence on the penetrance of oligogenic traits are frequently located in the coding genome (including coding variants in other complex disorders such as hypercholesteremia [73], and family-specific, low-penetrance variants associated with rare conditions such as oral clefts [74]) Exome Sequencing (ES) of all living, affected children in the iwi (J-5 to J-7 and I-1 to I-5) and their parents was performed, except for one parent where no DNA was available. Using this dataset, a susceptibility variant was hypothesized to have the following characteristics. These are listed in order of priority): (a) present in all affected members of the J-group (*n* = 4, with living, and DNA available for parents of J-4 to inform her possible genotypes), and at least 6 members of all affected iwi individuals (*n* = 9, including I-4 via parental genotypes); (b) rare global MAF (< 0.01) as reported by either the Exome Variant Server [75] or dbSNP (version hg19_137) [76]; (c) is phylogenetically conserved and/or there is a effect on a protein (predicted by SNPeff as a missense or protein-truncating variant); (d) rare (< 0.1 MAF) in Māori OR private to the iwi. The Māori frequency could only be estimated from the non-transmitted alleles of BA parents as no Māori genome-wide datasets are publically available; (e) affects a coding region of the genome (including + 2/− 2 bases from a splice-site boundary).

Exome coverage across all affected iwi individuals was above 10 reads for at least 97% of all targeted bases. No variants under a monoallelic or biallelic model of inheritance were identified that fulfilled all of the criteria listed above. Māori MAF, for which only limited information was available, was not used as an exclusionary factor as it did not distriminate between any variants already excluded by other means. No variant was observed in 5 or more individuals with BA, that exhibited at least moderate levels of phylogenetic conservation (scoring above 0.5 using phastCons [77]), and was predicted to be protein-altering in ANNOVAR [78]. Variants present in all available J-Group affected individuals were also examined (J-5, J-6, J-7, and either or both of J-4’s parents), with no requirement to be present in other samples. One variant fitted the remaining criteria, a known polymorphism in *ERAP1*, rs118132132, also carried by I-2. rs118132132 alters a highly conserved amino acid and is reported only in East-Asian populations [79], where it is present at a MAF of 1%. The absence of this candidate in 4/9 affected iwi individuals despite its relatively high frequency in East-Asian populations makes it implausible that it could explain this unusual BA cluster.

## Discussion

BA has a greater incidence in South-East Asian and Pacific populations compared to Europeans, but an elevated rate has only recently been rigorously demonstrated for New Zealand Māori. Due to the informal centralised referral of all cases of BA in New Zealand, a cluster of cases in the Bay of Plenty region was recognised, and multiple affected families identified reported distant relatedness to each other (the pedigree or J-group) or identified their lineage to the same two overlapping iwi. This iwi represents a unique opportunity to study BA in a cohort which likely shares a much stronger common genetic etiology. Taking the iwi and the pedigree as delineated groups, it was found that significantly more children with BA were born into these groups than would be expected in the Māori population not connected to the iwi - 6.8 times the rate of BA in the wider iwi and 50-fold in the pedigree. No unusual environmental factors were reported that were common to all or most affected children, many of whom were born in widely dispersed locations across the country. Seasonality was not found to be associated with BA as it has been in other populations.

Regional geographical clusters of BA have not been reported before, apart from a high rate reported in French Polynesia [14, 35] and a slight increase in one Scottish region [80], although the NZ iwi risk is an order of magnitude greater than the incidence in these areas. Of particular note is that all of the affected iwi children were born in the last quarter-century, suggesting a recently encountered environmental factor could have precipitated the development of BA in the presence of a genetic predisposition. Identifying environmental factors retrospectively is challenging, especially for a relatively small cohort, but studies are underway to study possible correlations with BA in the iwi.

The sporadic occurrence of BA within the iwi and pedigree makes it difficult to prioritise a genetic model to search for these hypothesised factors. Binary traits can occur both because of discrete, Mendelian factors or due to the presence of a liability threshold around which the additive risk from many loci leads to the expression of a phenotype. The study design pursued here can only identify discrete Mendelian factors, as additive risk contributed by many loci needs to be investigated with much larger sample sizes and is best pursued with a GWAS design.

In this iwi there was no evidence for elevated rates of homozygosity in most affected individuals whose parents had no known consanguinity, though high levels of homozygosity were noted generally in affected and unaffected Māori samples from both inside and outside of the iwi. This suggests that if a recessively-acting, high-risk factor is responsible for BA in this iwi, it is attributable to relatedness to common remote ancestors that is not discernible from the high rate of homozygosity in the Māori population more generally, at least using the genomic tools deployed here. This level of homozygosity is not unusual in populations with a recent history of founder effects, such as seen in present-day Finland [79, 80], Iceland [82] and the Greater Middle East [83]. For instance, similar levels of HBS have been seen in Finnish populations originating from founder groups 300–400 years ago [81], in which 90% of samples had runs of HBS > 5 cM.

Diseases that exist in a binary state (presence/absence of the disease) may be explained by the additive effect of many low-risk alleles [84], the genetics of which can be described using a liability threshold model. Given the sporadic occurrence of the disease, genetic factors with minor influence on the risk of BA are likely to be present in this cohort, similar to the *ADD3* SNP identified by previous GWAS [29, 30] with an odds ratio of 1.45 in European and East-Asian populations. Such minor risk factors may be transmitted by the relatively high-risk Māori background on which the disease cluster has occurred. It is possible that a confluence of these low-risk factors, already present in the general population, could explain a higher rate of the disease in one genetically connected group. However, a threshold model involving many loci would require an aggregation of many risk alleles in the iwi compared to the parental Māori population. These alleles would also have to be sufficiently numerous or of high enough risk, to increase the rate of BA by ~ 50-fold. The greater the number of low-risk alleles proposed by a threshold liability model, the greater the divergence between populations must be, and we found no evidence for this in this study. If a liability threshold model accurately applied in this instance, it would require some population history that distinguishes the iwi from other Māori, such as a founder effect coupled with reproductive isolation from other Māori. Alternatively there would need to be evidence for dramatic selection (such as for a geographically limited pathogen) acting across many loci, or significant admixture with an unidentified population carrying the risk alleles. Likewise, for a recessive factor, the spread of a causal allele from a very distant ancestor across the iwi would usually be accompanied by demonstrable reproductive isolation of the iwi to account for the 50-fold higher rate of BA in this community compared to the wider population. However, the history of the iwi and the area is indicative of frequent migrations, intermarriages and conflict between diverse groups over the past few hundred years [56, 57] and does not support the separation of the iwi from the Māori population. Supporting this historical data, PCA analysis (EIGENSOFT) and Bayesian clustering (STRUCTURE), found no evidence that the iwi demonstrated detectable genetic structure compared to the wider Māori population. If differential population structure was present between iwi and non-iwi Māori, the two groups would separate along the second eigenvector in Fig. 4, or a third population correlating with iwi identity would have been found with STRUCTURE (Fig. 4). However, there was no significant difference in the values of the two groups along this second eigenvector, and a hypothesis of only two populations (Māori and European) was shown to be the best hypothesis to explain the STRUCTURE data. This indicates there has been historic gene flow between the iwi and the wider Māori population. This is counter to a hypothesis of genetic isolation favouring an ancestral recessive variant specific to the iwi, or multiple risk variants, leading to susceptibility to develop BA.

An alternative model that was considered here was a factor inherited from a relatively recent common ancestor with the low-penetrance of this factor and/or dependence on environmental modifiers invoked to explain the relatively recent appearance of the phenotype. Using Exome Sequencing (ES), no clear candidate variants were identified under either a dominant or recessive model within the coding genome to support such a model. Further investigation to define which regions of the genome the affected individuals share may reduce the search space for this putative susceptibility conferring factor. Relevant genetic elements that could not be considered as potentially predisposing to the development of BA in this study include intergenic variants, which are not captured for sequencing by the exome approach used here but can effect expression and regulation of one or more genes, and large structural variants that may not be detected with short-read sequencing but could change the copy number of one or more genes. The cluster of BA cases in this iwi represents a globally and historically unique chance to further understanding of the etiology of BA.

## Conclusions

We presented a cluster of biliary atresia cases that to our knowledge is unique in the literature, with up to 50 times the incidence of the disease compared to an already elevated rate in an indigenous, understudied New Zealand Māori population. Despite a complicated population history of bottlenecking and spare consanguinity, we found no evidence of elevated homozygosity across most of the affected children, nor evidence that the affected iwi had a history of reproductive isolation from the wider Māori population. We proposed that a dominant, low-penetrance variant in combination with environmental factors could explain this pattern of biliary atresia, possibly inherited by most of the affected children within the two centuries spanned by their pedigree. However, Exome Sequencing of all available affected individuals did not uncover a plausible candidate variant under a mono- or biallelic model, and further research is required, such as investigating alternative categories of variation such a copy number.

## Additional files

Additional file 1: **Table S1.** Estimates of the number of extant descendents of Couple J (DOCX 80 kb)

## Abbreviations

ANOVA: Analysis of Variance
BA: Biliary Atresia
BWA: Burrows-Wheeler Aligner
CI: Confidence Interval
cM: centiMorgans
GATK: Genome Analysis Toolkit
GWAS: Genome Wide Association Study
HBS: Homozygosity-by-state
IBD: Identity-by-descent
IBS: Identity-by-state
NZ: New Zealand
PCA: Principal component analysis
RR: Relative Rate
WES: Whole Exome Sequencing
