# Supplementary material for: Genetic investigation into an increased susceptibility to biliary atresia in an extended New Zealand Māori family
Source: BMC Med Genomics. 2018 Dec 18;11:121. doi: 10.1186/s12920-018-0440-0 (PMC6299523; doi:10.1186/s12920-018-0440-0)
Supplement: Supplementary file 1 — Table S1. Estimates of the number of extant descendents of Couple J (DOCX 80 kb) [file 12920_2018_440_MOESM1_ESM.docx]

Supplementary Materials

**Additional file 1: Table S1. Estimates of the number of descendants each generation from Couple J**

| Year | Generation | Māori population increase | Fertility increase | No Consanguinity | 1/3 Consanguinity from 4th gen |
| --- | --- | --- | --- | --- | --- |
| 1851 | 1 | - | - | 4 | 4 |
| 1876 | 2 | 1 | 1 | 8 | 8 |
| 1901 | 3 | 1 | 1 | 16 | 16 |
| 1926 | 4 | 1 | 1 | 32 | 27 |
| 1951 | 5 | 2 | 2 | 128 | 106 |
| 1976 | 6 | 3 | 2 | 512 | 425 |
| 2001 | 7 | 1 | 1 | 1024 | 850 |

Description of Columns in Table S1:

**Year –** calendar year at the beginning for each generation; the first year is taken from the birth year of Couple J’s eldest child known to have survived to adulthood

**Māori Population Increase** – the factor by which the Māori population is estimated to have increased from the previous generation. The final count is 1 despite a higher-than-replacement birthrate because a full generation has not passed yet.

**Fertility increase -** the factor by which the fertility is estimated to have increased for Māori woman over the previous generation (estimated from both census data where available and population sizes where direct fertility data was not available)

**No Consanguinity** – the estimated number of children born in each generation if all marriages are outbred (ie, to people who are not descended from Couple J)

**1/3 Consanguinity from 4^th^ Gen –** the estimated number of children born in each generation if the number of reported marriages to other Group J descendants is proportional to the estimated J-group population.
